# Emotion and Psychophysiological Responses During Emotion–Eliciting Film Clips in an Eating Disorders Sample

**DOI:** 10.3389/fpsyg.2021.630426

**Published:** 2021-07-08

**Authors:** Melanie N. French, Eunice Y. Chen

**Affiliations:** ^1^Temple Eating Disorders Program, Department of Psychology, Temple University, Philadelphia, PA, United States; ^2^Department of Psychiatry and Behavioral Neurosciences, University of Chicago, Chicago, IL, United States

**Keywords:** emotion, affect, binge eating, bulimia, anorexia nervosa, respiratory sinus arrhythmia, galvanic skin response, eating disorders

## Abstract

**Background:** Greater vulnerability to negative emotions appears associated with the development and maintenance of eating disorders (EDs). A systematic review of psychophysiological studies using emotion-eliciting film clips reveals that there are no studies examining the effect of standardized validated film clips on psychophysiological response across a range of EDs.

**Methods:** Using standardized validated film clips without ED-specific content, the present study examined self-reported emotions and psychophysiological responses of women with Binge-Eating Disorder (BED; *n* = 57), Anorexia Nervosa (AN; *n* = 16), Bulimia Nervosa (BN; *n* = 34), and Healthy Controls (HCs; *n* = 26) at Baseline, during Neutral, Sad, Happy, and Fear-inducing film clips, and at Recovery.

**Results:** Throughout the protocol, the ED groups reported significantly greater sadness and anxiety than HCs. Additionally, the AN group reported more fear, the BED group more frustration, and the BED and BN groups more tension than HCs. Compared to HCs, the BED group reported stronger urges to binge throughout the protocol, whereas BN group reported stronger urges to binge relative to the HC group only at Baseline and Recovery. The BN and BED groups experienced decreased urges to binge during all film clips compared to Baseline. Respiratory Sinus Arrhythmia levels were significantly lower in the BED group compared to HCs and the BN group throughout the protocol.

**Discussion:** Standardized validated film clips can be used to elicit expected self-reported emotion and skin conductance responses in ED groups, although individuals with EDs compared HCs report greater negative emotions. Interestingly, film clips appeared to reduce urges to binge in binge-eating groups.

## Introduction

Multiple theories have pointed to negative affect as a trigger for engagement in disordered eating. In response to negative affect, models suggest that eating disorder behaviors, such as binge-eating or restriction, may serve as maladaptive emotion regulation strategies (Heatherton and Baumeister, 1991; Polivy et al., 1994; Hohlstein et al., 1998; Corstorphine, 2006; Haedt-Matt and Keel, 2011; Gross and Jazaieri, 2014). However, there are limitations in the research regarding our understanding of emotional processing in eating disorder (ED) samples. Most studies do not induce emotions in a way that has been is both experimentally manipulated and has high ecological validity. Past methods of inducing affect include the use of slides (e.g., Lazarus et al., 1962), hypnosis (e.g., Bower et al., 1983), interactions with confederates (e.g., Ax, 1953), and music (e.g., Sutherland et al., 1982). In order to address the limitations of past research methods, Gross and Levenson (1995) and other research teams (Schaefer et al., 2010) validated films clips designed to elicit discrete emotions in a time-efficient, dynamic, and ecologically valid manner.

Studies measuring facial expressions and self-reported emotions after film mood inductions show individuals with Anorexia Nervosa (AN) tend to have an inhibited emotional facial expression of both positive and negative emotions (Davies et al., 2016), despite reporting similar or more intense emotions (Davies et al., 2011, 2013; Rhind et al., 2014; Dapelo et al., 2015; Lang et al., 2016). Some evidence shows individuals with Bulimia Nervosa (BN) may experience blunted affect (Claes et al., 2012), whereas other studies found BN samples had similar emotional response to Healthy Controls (HCs; Dapelo et al., 2015). For individuals with Binge-eating Disorder (BED), there is evidence that negative emotions precede binges; however, there is lack of causal data to support binge-eating as a function of negative affect (Dingemans et al., 2017).

Emotions have a psychophysiological component (Barrett et al., 2007) and measures of heart rate indices and skin conductance (SC) can provide insight into this aspect of emotional response. Respiratory sinus arrhythmia (RSA), a measure of heart rate variability throughout the respiration cycle, is associated parasympathetic function, while skin conductance is related to sympathetic activity (Boucsein, 1992; Beauchaine and Thayer, 2015).

We conducted a systematic review of film mood induction studies that also assessed RSA and/or SC in samples with disordered eating, which yielded only three relevant studies. One study using a body-related clip mood induction found an increase in negative affect (i.e., sadness and anxiety), desire to binge, and SC fluctuations in a BED group but found no changes in the HC group (Svaldi et al., 2009). Another study showed abnormalities in parasympathetic activity in an AN group (restricting type) compared to HCs at baseline and in response to a negative film mood induction (Rommel et al., 2015). Finally, another study found individuals with BN experienced more negative emotions than restrained eaters or HCs after a negative film mood induction (Tuschen-Caffier and Vogele, 1999). Although restrained eaters showed greater skin conductance levels (SCL) than the BN and HC groups, these results were not replicated for other sympathetic measures, such as heart rate. See https://osf.io/8vxen/?view_only=73da0b3db29c47558f74133e8112f3a3 for systematic review details. To our knowledge, there is no study that measures self-reported and psychophysiological data using multiple film mood inductions (e.g., fear, sadness, or happiness) across ED groups in a single study.

The present study measures RSA, SC, and self-reported emotions at baseline, and after film clips validated to elicit either a neutral, sad, happy, or fearful emotional state, and a recovery period in women with AN, BED, and BN as well as HCs. We hypothesized when compared to HCs, (1) women with AN, BED, and BN would have more reported negative emotions, fewer positive emotions, and greater urges to binge (particularly the BED and BN groups) throughout the protocol, and especially after the sad and fear film clips. Understanding the specific emotions that trigger disordered eating behaviors may inform treatment. In addition, by measuring urges to binge, we can investigate the underexplored question on the causal relationship between urges to binge and emotion. In terms of psychophysiological findings, we predicted that (2) women with EDs would have lower average RSA compared to HCs. Finally, we expected to see (3) more skin conductance responses (SCRs) and greater tonic SCLs after each emotion-inducing film clips compared to the neutral film clip.

## Materials and Methods

### Participants

The sample consisted of 57 women who met criteria for BED, 16 for AN, and 34 for BN and 26 HCs. Participants with EDs were recruited from EDs treatment studies at a university hospital, and HCs were recruited from the community using flyers and online postings. Prior to starting the study, all measures and procedures were approved by the Institutional Review Board, and participants provided informed consent. In an initial session, a Masters-level clinician administered the Eating Disorders Examination-16 (Fairburn et al., 2008) and the Structured Clinical Interview for Diagnostic and Statistical Manual of Mental Disorders–IV-Text Revision [DSM-IV-TR; (American Psychiatric Association, 2000; First et al., 2002)]. The second session consisted of the experimental procedure. See Supplementary Material 1 for full Inclusion and Exclusion criteria.

### Procedure

Participants were assessed prior to any treatment (see Table 1 for the procedure). At Baseline, self-reported emotions were first collected, followed by a 5-min “true” baseline, where participants were asked to “relax without focusing on anything in particular” while recording RSA and SC. Then, the four film clips were presented in random order across subjects. RSA and SC were assessed during each of the film clips. After each film clip, self-reported emotions were assessed, followed by psychophysiological recordings during a “Active” baseline where participants are asked to count the number of times a given color appears on the computer screen (called “Vanilla baseline” in Jennings et al., 1992). This task was designed to reduce return emotions to a neutral state in order to reduce carry-over effects from viewing previous film clip stimuli. During the Recovery period, self-reported emotions are assessed a final time, following by recordings at “active” baseline.

**Table 1:** Study procedure timeline.

**Study procedure timeline**	
**Baseline**	
VAS of emotions assessment	1 min
“True” baseline	5 min
**Sad clip (The Champ)**	
Film clip shown^†^	3 min
VAS of emotions assessment	1 min
“Active” baseline	3 min
**Happy clip (When Harry Met Sally)**	
Film clip shown^†^	3 min
VAS of emotions assessment	1 min
“Active” baseline	3 min
**Fear clip (Silence of the Lambs)**	
Film clip shown^†^	3 min
VAS of emotions assessment	1 min
“Active” baseline	3 min
**Neutral clip (Denali documentary)**	
Film clip shown^†^	3 min
VAS of emotions assessment	1 min
“Active” baseline	3 min
**Recovery period (after last clip)**	
“Active” baseline	5 min
VAS emotions assessment	1 min

† *Film clips were presented in a random order per subject. “True” baseline and “Active” baseline involved assessment of respiratory sinus arrhythmia and skin conductance*.

*Min, minute; VAS, visual analog scale*.

### Film Clip Stimuli

Participants viewed four 3-min film clips on a computer screen in a randomized order to elicit either a positive emotion—happiness (“When Harry Met Sally”), sadness (“The Champ”), fear (“The Silence of the Lambs”), or a neutral mood (“Denali”). Clips were previously validated (Gross and Levenson, 1995). The film stimuli did not have an ED-specific content, such as topics related to food or body image.

### Measures

The Eating Disorders Examination (EDE) Version 16.0 is a standardized semi-structured interview, measuring the frequency and severity of ED psychopathology (Fairburn et al., 2008). It has high internal consistency, inter-rater reliability, and test-retest reliability (Rizvi et al., 2000).

The Structured Clinical Interview for DSM–IV–TR (SCID) is a standardized semi-structured clinical interview used to assess psychological disorders with adequate inter-rater reliability (American Psychiatric Association, 2000; First et al., 2002).

Participants reported their current emotional states using a Visual Analog Scale [VAS; (Haines et al., 1995)] at: (1) Baseline, and after the (2) neutral film clip (termed Neutral clip), the (3) sadness-inducing film clip (Sad clip), (4) happiness-inducing film clip (Happy clip), and (5) fear-inducing film clip (Fear clip), and (6) completion of the task (i.e., Recovery). The VAS ranged from 0 to 100 and assessed frustration, anxiety, happiness, tension, fear, sadness, and “urges to binge,” with higher VAS scores indicating greater emotional intensity.

### Psychophysiological Measures

We collected electrocardiogram data utilizing a modified Lead II configuration. We derived RSA by using a band pass filter on the electrocardiogram signal and spectral analysis to extract the high-frequency component (>0.15 Hz) of heart rate variability. We measured SC by using two electrodes placed on the palm of the non-dominant hand. Tonic SCL refers to level SC collected over each period, whereas SCRs refer to the number of responses per period.

### Statistical Treatment

Preliminary analyses included an assessment of group differences in demographic and clinical characteristic variables performed using ANOVAs or Chi-square (χ^2^) for continuous and non-continuous variables, respectively. *Post-hoc* tests were conducted using simple contrasts for ANOVAs or adjusted residuals for Chi-square tests when significant group differences were found.

Repeated measures ANOVAs during six Conditions: Baseline, Neutral clip, Sad clip, Happy clip, Fear clip, and during Recovery, by four Group variables: BN, BED, AN, and HC were conducted. ANOVAs examined separately for self-reported anxiety, fear, frustration, sadness, tension, happiness, and urges to binge, as well as RSA, SCR, and SCL, resulting in 10 separate ANOVAs. A result was considered statistically significant if *p* < 0.05. SPSS Statistics 26 (IBM Corp. Released 2019. IBM SPSS Statistics for Windows, Version 26.0. Armonk, NY: IBM Corp) was used. *Post-hoc* comparisons were corrected using a Bonferroni adjustment. The output of the results may be found in https://osf.io/8vxen/?view_only=73da0b3db29c47558f74133e8112f3a3.

## Results

### Sample Description

There were no significant group differences on marital status or full-time employment/student status. The AN group relative to the other groups had more individuals who identified as mixed-race (*p* = 0.042). Individuals with BED were older than individuals with BN and HCs (*p*'s < 0.001). The AN group and HCs were more likely to be low-income (i.e., annual income below $25,000; *p*'s < 0.03) while the BED group was less likely to be low-income (*p* < 0.001). Older age and higher income were correlated (*r*=0.343; *p* < 0.001). Table 2 reports the sociodemographic group characteristics. Supplementary Table 2 shows a correlation matrix with the demographic variables.

**Table 2:** Sociodemographic and clinical characteristics of the sample.

	**Anorexia nervosa**	**Binge eating disorder**	**Bulimia nervosa**	**Healthy controls**		
	***n* = 16**	***n* = 57**	***n* = 34**	***n* = 26**		
	**M (SD)**	**M (SD)**	**M (SD)**	**M (SD)**	**F(df)**	***p*-value**
Age in years	35.75 (11.17)	41.00 (10.18)	29.47 (8.24)	28.65 (10.63)	14.00 (3,129)	<0.001
BMI (kg/m^2^)^†^	18.66 (3.45)	36.72 (10.76)	25.00 (5.31)	26.38 (4.48)	30.77 (3,129)	<0.001
EDE-EC^†^^,^^‡^	3.10 (1.99)	2.72 (1.36)	2.78 (1.17)	0.02 (0.07)	32.24 (3,128)	<0.001
EDE-DR^†^^,^^‡^	3.29 (2.02)	2.18 (1.24)	3.06 (1.37)	0.44 (0.88)	22.70 (3,128)	<0.001
EDE-WC^†^^,^^‡^	2.61 (1.89)	3.61 (1.08)	3.40 (1.40)	0.53 (0.77)	38.52 (3,128)	<0.001
EDE-SC^†^^,^^‡^	3.27 (1.81)	3.81 (1.22)	3.65 (1.36)	0.50 (0.77)	42.79 (3,128)	<0.001
Objective binge episodes^§^	1.22 (2.38)	4.26 (3.99)	6.42 (6.24)	0.00 (0.00)	13.73 (3,129)	<0.001
Vomit episodes^§^	2.22 (3.94)	0.13 (0.93)	7.60 (8.34)	0.00 (0.00)	22.74 (3,129)	<0.001
	***N*** **(%)**	***N*** **(%)**	***N*** **(%)**	***N*** **(%)**	**x**^**2**^	***p*****-value**
Caucasian	13 (81.25)	39 (68.42)	24 (70.59)	15 (57.69)	21.48 Δ (3,129)	0.044
Black	0 (0.00)	8 (14.04)	3 (8.82)	5 (19.23)		
Asian	0 (0.00)	2 (3.51)	2 (5.88)	3 (11.54)		
Mixed	3 (18.75)	6 (10.53)	0 (0.00)	0 (0.00)		
Hispanic	0 (0.00)	2 (3.51)	5 (14.71)	3 (11.54)		
American Indian/Alaska Native	0 (0.00)	0 (0.00)	0 (0.00)	0 (0.00)		
Native hawaiian or other pacific islander	0 (0.00)	0 (0.00)	0 (0.00)	0 (0.00)		
Marital status: single^‡^	9 (75.00)	25 (44.64)	20 (60.61)	17 (68.00)	6.44 (3,122)	0.092
Employment status^‡^^,^^||^	14 (87.50)	54 (94.74)	30 (90.91)	25 (96.15)	4.60 (3,128)	0.596
Income status^‡^^,^^¶^	12 (75.00)	16 (28.07)	18 (54.55)	18 (72.00)	20.02 (3,127)	<0.001
Mood disorders	7 (43.75)	35 (61.40)	25 (73.53)	1 (3.85)	32.85 (3,129)	<0.001
Anxiety disorders	10 (62.50)	20 (35.09)	10 (29.41)	0 (0.00)	19.87 (3,129)	<0.001
Medical comorbidities^‡^	8 (61.54)	25 (47.17)	21 (75.00)	4 (23.53)	12.43 (3,107)	0.006
Medication use	6 (37.50)	29 (50.88)	16 (47.06)	2 (7.69)	14.88 (3,129)	0.002

† *BMI, Body Mass Index, EDE, Eating Disorder Examination; EC, Eating Concerns; DR, Dietary Restraint; WC, Weight Concerns; SC, Shape Concerns*.

‡ *Missing participants' responses for: EDE scales (1 HC); Marital status (4 AN, 1 BED, 1 BN, 1 HC); Employment status (1 BN); Income status: (1 BN, 1 HC); and Medical comorbidity (3 AN, 4 BED, 6 BN, 9 HC)*.

§ *Within the previous three months*.

|| *Employment status = Employment/Full-time student status*.

¶ *Income status = low income status i.e., household income is < $25 000*.

*ΔValues for racial group differences*.

Overall, group differences in clinical characteristics aligned with diagnosis status (see Table 2). As expected, the BED group had the highest BMI, followed by the HC and the BN groups, while the AN group had the lowest BMI. All group differences for BMI were significant (*p's* < 0.001) except for the BN and HC group comparison. ED groups had greater ED psychopathology, as measured by the EDE subscales (*p's* < 0.001). Additionally, the BN group also scored higher than the BED group on the dietary restraint EDE subscale (*p* = 0.003). Mood disorders, anxiety disorders, medical comorbidities, and use of medical services were less frequently reported by the HC group (*p's* < 0.001). The BED and BN groups reported more mood disorders (*p* = 0.040 and 0.002, respectively); the AN group reported more anxiety disorders (*p* < 0.001). The BED group reported a higher rate of medication use (*p* = 0.024), while the BN group had more medical problems, such as high blood pressure and cholesterol (*p* = 0.005). Consistent with clinical presentation, the BED and BN groups higher rates of binging than the AN and HC groups (*p's* < 0.001). Finally, individuals with BN reported the highest frequency of vomiting episodes compared to all other groups (*p's* < 0.004), and individuals with AN reported more vomiting episodes than HCs (*p* = 0.040).

### Main Analyses

#### Negative Self-Reported Emotions

See Table 3 for means and standard deviations of the negative emotion variables. There were significant main effects of Condition for anxiety [*F*_(5, 97)_ = 37.52, *p* < 0.001, η^2^ = 0.279], fear [*F*_(5, 97)_ = 52.90, *p* < 0.001, η^2^ = 0.353], frustration [*F*_(5, 97)_ = 14.76, *p* < 0.001, η^2^ = 0.132], sadness [*F*_(5, 97)_ = 68.06, *p* < 0.001, η^2^ = 0.412], and tension [*F*_(5, 97)_ = 46.78, *p* < 0.001, η^2^ = 0.0.325]. Anxiety, fear, and tension were higher after the Fear clip, compared to all other clips (*p's* < 0.05). Sadness was rated the highest after the Sad clip compared to all other clips (*p's* < 0.001).

**Table 3:** Self-reported emotions, urge to binge, respiratory sinus arrhythmia, and skin conductance measures for groups at baseline, after each film clip, and during recovery.

	**Baseline**	**Neutral clip**	**Sad clip**	**Happy clip**	**Fear clip**	**Recovery**
	**M (SD)**	**M (SD)**	**M (SD)**	**M (SD)**	**M (SD)**	**M (SD)**
**Anxious emotion**						
AN (*n =* 10)	56.73 (22.14)	10.30 (18.51)	46.65 (25.81)	25.02 (27.00)	60.17 (11.31)	37.92 (29.82)
BED (*n =* 47)	41.30 (29.13)	11.95 (17.06)	40.51 (29.26)	13.26 (19.95)	56.65 (29.18)	34.28 (27.68)
BN (*n =* 27)	47.99 (28.96)	9.85 (18.57)	32.25 (31.06)	17.83 (27.43)	54.51 (33.02)	25.83 (27.20)
HCs (*n =* 17)	12.25 (22.07)	4.42 (12.14)	27.31 (27.24)	9.92 (18.47)	40.13 (30.31)	13.71 (20.64)
**Fear emotion**						
AN (*n =* 10)	42.42 (29.20)	0.95 (1.93)	28.42 (26.88)	20.88 (27.08)	59.92 (21.19)	22.47 (29.58)
BED (*n =* 47)	25.41 (25.44)	10.30 (16.01)	27.74 (29.83)	5.50 (11.15)	51.40 (28.47)	11.88 (20.26)
BN (*n =* 27)	30.65 (27.39)	5.91 (12.55)	30.44 (32.31)	8.55 (18.93)	54.84 (35.91)	8.64 (15.06)
HCs (*n =* 17)	4.27 (10.47)	1.72 (4.71)	22.03 (27.60)	3.01 (6.35)	36.41 (29.42)	6.41 (14.32)
**Frustration emotion**						
AN (*n =* 10)	39.35 (31.20)	7.87 (14.44)	29.25 (30.25)	14.20 (21.97)	24.85 (25.13)	35.38 (33.79)
BED (*n =* 47)	35.10 (29.45)	15.73 (21.78)	30.99 (29.52)	11.50 (18.69)	38.66 (32.02)	38.91 (31.24)
BN (*n =* 27)	32.27 (26.84)	12.44 (21.08)	25.44 (27.59)	9.74 (16.26)	27.84 (28.39)	36.06 (27.91)
HCs (*n =* 17)	7.30 (13.05)	4.51 (11.14)	22.49 (26.07)	8.35 (15.20)	24.45 (26.89)	13.92 (21.32)
**Sadness emotion**						
AN (*n =* 10)	42.75 (20.95)	14.80 (22.89)	71.12 (12.11)	16.68 (22.02)	22.38 (22.25)	19.85 (19.66)
BED (*n =* 47)	36.48 (28.66)	14.74 (21.26)	66.92 (30.62)	8.87 (14.35)	27.50 (26.64)	21.63 (23.50)
BN (*n =* 27)	37.22 (29.89)	14.19 (25.38)	67.14 (29.92)	6.00 (13.96)	28.09 (30.51)	22.27 (29.92)
HCs (*n =* 17)	5.65 (11.79)	4.73 (14.17)	51.32 (32.33)	2.13 (4.83)	16.19 (22.43)	10.53 (18.79)
**Tension emotion**						
AN (*n =* 10)	52.95 (27.55)	8.90 (15.64)	47.88 (27.76)	23.20 (23.94)	64.05 (11.18)	43.95 (29.65)
BED (*n =* 47)	48.72 (27.06)	15.76 (20.00)	49.98 (30.08)	15.35 (19.13)	60.94 (28.91)	39.34 (29.28)
BN (*n =* 27)	51.97 (29.32)	11.64 (23.05)	43.66 (34.82)	25.20 (29.97)	60.63 (35.57)	34.18 (32.42)
HCs (*n =* 17)	18.40 (19.63)	6.60 (15.16)	31.25 (31.55)	13.28 (21.49)	48.49 (32.28)	16.60 (23.58)
**Happiness**						
AN (*n =* 10)	40.70 (23.84)	46.83 (19.86)	5.92 (9.10)	57.58 (27.61)	15.70 (23.73)	29.75 (21.57)
BED (*n =* 47)	41.45 (17.60)	56.09 (25.91)	11.40 (17.16)	66.33 (20.91)	18.20 (21.26)	35.95 (22.19)
BN (*n =* 27)	43.48 (17.61)	47.90 (24.96)	8.83 (14.78)	58.01 (27.43)	15.84 (23.61)	32.15 (24.62)
HCs (*n =* 17)	59.89 (20.43)	55.89 (28.48)	13.88 (16.95)	61.32 (25.13)	23.32 (24.14)	52.61 (22.55)
**Urge to binge**						
AN (*n =* 10)	25.55 (28.80)	5.40 (15.69)	8.90 (22.94)	15.15 (24.05)	12.38 (26.20)	20.13 (28.97)
BED (*n =* 47)	42.30 (27.39)	20.15 (23.92)	29.36 (29.89)	23.63 (27.12)	25.75 (29.80)	35.43 (30.43)
BN (*n =* 27)	36.06 (29.26)	9.75 (19.42)	12.57 (23.01)	11.07 (20.69)	13.12 (24.03)	23.83 (25.97)
HCs (*n =* 17)	0.86 (2.07)	0.45 (1.15)	0.63 (2.05)	0.64 (1.58)	0.54 (1.48)	0.71 (1.91)
**Respiratory sinus arrhythmia**						
AN (*n =* 16)	5.91 (1.17)	5.78 (1.20)	5.66 (1.36)	5.66 (1.13)	5.76 (1.13)	6.03 (1.36)
BED (*n =* 57)	5.29 (1.58)	5.24 (1.35)	5.10 (1.65)	5.26 (1.53)	5.17 (1.34)	5.25 (1.36)
BN (*n =* 34)	6.38 (1.10)	6.24 (0.96)	6.01 (1.23)	6.11 (1.03)	6.09 (1.12)	6.46 (1.00)
HCs (*n =* 26)	6.23 (1.41)	6.38 (1.26)	6.36 (1.27)	6.30 (1.21)	6.18 (1.45)	6.32 (1.21)
**Skin conductance response**						
AN (*n =* 12)	2.40 (2.81)	2.54 (1.92)	3.78 (5.23)	3.47 (3.47)	3.29 (2.78)	3.28 (3.10)
BED (*n =* 44)	2.75 (2.31)	2.16 (1.89)	2.70 (2.57)	3.95 (2.84)	3.52 (2.33)	3.18 (2.17)
BN (*n =* 24)	1.59 (1.89)	2.29 (2.68)	2.32 (2.28)	3.78 (3.98)	2.53 (2.28)	3.65 (4.03)
HCs (*n =* 21)	1.86 (1.71)	1.48 (1.33)	2.37 (2.58)	2.46 (2.22)	2.13 (2.76)	2.99 (2.91)
**Skin conductance level**						
AN (*n* = 11)	3.14 (2.78)	3.64 (3.53)	5.66 (7.09)	4.54 (4.33)	4.77 (4.31)	4.91 (4.61)
BED (*n =* 43)	3.61 (2.74)	3.88 (3.02)	4.03 (3.42)	4.05 (3.15)	4.35 (2.87)	4.11 (3.10)
BN (*n =* 24)	3.00 (4.16)	4.28 (5.47)	4.84 (6.38)	4.90 (5.17)	3.93 (4.43)	4.94 (5.55)
HCs (*n =* 21)	2.32 (3.12)	2.95 (3.25)	5.35 (9.10)	4.44 (5.54)	3.97 (5.56)	4.96 (6.75)

*Data was excluded from 32 participants in emotion self-report variables and skin conductance response and 34 participants for skin conductance level due to missing data during at least one condition*.

*AN, Anorexia Nervosa; BED, Binge-Eating Disorder; BN, Bulimia Nervosa; HCs, Healthy Controls*.

For self-reported anxiety [*F*_(3, 97)_ =5.18, *p* = 0.002, η^2^ = 0.138], fear [*F*_(3, 97)_ = 3.46, *p* = 0.019, η^2^ = 0.097], frustration [*F*_(3, 97)_ =3.33, *p* = 0.023, η^2^ = 0.093], sadness [*F*_(3, 97)_ = 4.46, *p* = 0.006, η^2^ = 0.121], and tension [*F*_(3, 97)_ =3.61, *p* = 0.016, η^2^ = 0.101], there was also a main effect of Group. ED groups reported anxiety and sadness as more intense than HCs (*p's* < 0.05). Additionally, compared to HCs, the AN group reported more fear (*p* = 0.023), the BED group more frustration (*p* = 0.013), and the BED and BN groups more tension than HCs (*p's* < 0.05). There were no significant interaction effects.

#### Positive Emotion

For happiness, there was an effect of Condition [*F*_(5, 97)_ = 80.82, *p* < 0.001, η^2^ = 0.455], where happiness had the highest ratings after the Happy clip compared to all conditions except the neutral clip (*p's* < 0.001). There was not a significant effect of Group or a Group by Condition interaction. See Table 3 for means and standard deviations for the happiness rating.

#### Urge to Binge

For urge to binge, there was a main effect of Condition [*F*_(5, 97)_ = 11.25, *p* < 0.001, η^2^ = 0.104] and a main effect of Group [*F*_(3, 97)_ = 9.89, *p* < 0.001, η^2^ = 0.234]; however, Group and Condition effects were qualified by an interaction between Group and Condition [*F*_(15, 97)_ = 1.72, *p* = 0.043, η^2^ = 0.051]. Simple effects were significant for all Conditions between Groups (*p's* < 0.006). The BED group reported stronger urges to binge compared to: (1) the HC group during all of the Conditions (*p's* < 0.02) and (2) the BN group after the Sad film clip (*p*'s = 0.037). *Post-hoc* tests showed that the BN group reported greater urges to binge than the HC group only at Baseline and Recovery but not after the film clips (*p's* < 0.04).

Simple effects examined for all Groups between Conditions showed that there were significant differences for the BN and BED groups (*p's* < 0.001). BN and BED groups experienced stronger urges to binge at Baseline compared to all film conditions (*p's* < 0.005). In the BED group, urges to binge were stronger (1) during Recovery than after the Neutral and Happy clips (*p's* < 0.03) and (2) after the Sad film clip compared to the Neutral film clip (*p* = 0.013). No simple effect differences in response to Conditions were observed for the AN and HC groups. See Table 3 for means and standard deviations of the Urge to binge variable.

#### Respiratory Sinus Arrhythmia

There was a main effect of Condition [*F*_(5, 129)_ = 4.40, *p* = 0.001, η^2^ = 0.033], where average RSA was lower during the Fear and Sad clips compared to the during Recovery (*p's* < 0.03). There was also a significant main effect of Group [*F*_(3, 129)_ = 6.62, *p* < 0.001, η^2^ = 0.133], such that individuals with BED exhibited significantly lower average RSA levels than individuals with BN and HCs (*p's* < 0.003) at all conditions. There was no interaction between Condition and Group. See Table 3 for means and standard deviations of RSA.

#### Skin Conductance

There was a significant main effect of Condition for average number of SCRs [*F*_(5, 97)_ =6.08, *p* < 0.001, η^2^ = 0.059] and average tonic SCL [*F*_(5, 96)_ = 6.41, *p* < 0.001, η^2^ = 0.063]. After the Neutral clip, average SCRs and SCLs was reduced compared to during Recovery (*p's* < 0.005). In addition, after the Neutral clip there were fewer average SCRs compared to after the Happy and Fear clips (*p's* < 0.02). There were fewer average SCRs and lowered average SCLs during Baseline than after the Happy clip and during Recovery (*p's* < 0.03). Additionally, there was also reduced average SCLs during Baseline than after the Sad and Fear clips (*p's* < 0.02). There were no effects of Group or interaction effects for either average SCRs or tonic SCLs. See Table 3.

## Discussion

Standardized validated film clips, presented in a random order, specifically elicited intended emotions and average SCL and SCR responses across all groups. We found that individuals with EDs exhibited more negative emotions than HCs throughout the protocol. Interestingly, both the BN and BED groups had higher urges to binge at Baseline compared to the film clip conditions. Our psychophysiological findings show that the BED group had lower average RSA levels than HCs or the BN group.

### Self-Reported Emotions

Taken together, our findings indicated individuals with EDs exhibit stronger negative emotions, especially sadness and anxiety, compared to HCs. There was no interaction between Group and Condition, supporting the idea that EDs have consistently higher negative emotional state, but not necessarily greater emotional reactivity to negative emotion-eliciting stimuli, at least when the stimuli is not ED-specific. This is consistent with some studies that showed ED groups report negative baseline emotion responses, but were *not* more reactive to emotion-eliciting stimuli using sad film clips than HCs (Danner et al., 2013, 2016; Naumann et al., 2016). Together with these recent studies, our findings challenge the theoretical hypothesis that general negative affect and increased emotional reactivity contribute to the maintenance of disordered eating, where disordered eating becomes a maladaptive emotion regulation strategy (Polivy et al., 1994; Haedt-Matt and Keel, 2011; Haynos and Fruzzetti, 2011). In contrast, ED samples may experience more negative emotion reactivity to disorder-specific stimuli, such as food (Ferrer-Garcia and Gutiérrez Maldonado, 2005; Christensen et al., 2020).

### Urge to Binge

Our results add to the literature in examining urges to binge after the presentation of validated, emotion-eliciting film clips in women with different EDs relative to HCs. The pattern of urges to binge for the different groups did not fit the pattern of self-reported negative emotions. The BED group had stronger urges to binge compared to the HCs throughout the protocol, and the BN group had stronger urges to binge compared to HCs at Baseline and Recovery. Interesting, for both the BED and BN groups, urges to binge were *higher* during Baseline than after both positive and negative emotion-eliciting film clips. This is contrary to other studies using ecological momentary assessment (EMA) that have shown that urges to binge are preceded by negative emotions, although there has been limited experimental evidence showing a causal relationship between negative affect and urges to binge (see Dingemans et al., 2017 for a review). EMA studies are observational and not experimental but are conducted in real-life settings rather than laboratory settings. These differences between EMA and experimental studies may have contributed to the discrepancy between our findings and studies using EMA. Svaldi et al. (2010) found women with BED had an increased desire to binge after watching a sad film without instructions, however, it is unclear whether standardized validated film clips were used in this study. Another study in a sample with obesity and binge-eating problems found no effect on film type on “urge to eat” (Chua et al., 2004). A recent meta-analysis found no effect between negative affect using mood induction procedures and food consumption for individuals with eating disorders, however, it is important to note that none of the studies included validated film clips as the mood induction procedure (Evers et al., 2018). Choice of the word “binge” instead of “hunger” or “eat” on the VAS scale assessing “urge to binge” may have increased the likelihood of stronger responses by the BED and BN group than HC groups and further studies are needed to explore this. Further exploration of the mechanism as to why individuals with BED and BN had stronger urges to binge prior to and after emotion-inducing films and why urges to binge in these groups were lower after the emotion-inducing film clips is needed.

### Discrepancies Between Psychophysiological Results and Self-Reported Emotions

Although individuals with BED had lower average RSA levels throughout the protocol, there were no interaction effects or any other significant group differences among our psychophysiological findings. Lower resting RSA levels have been associated with a greater number of psychological problems, including dysregulated emotions and greater negative affect (Carney et al., 2001; Crowell et al., 2017; Sloan et al., 2017), but patterns have been less consistent when looking at emotional reactivity. Previous psychophysiological experiments using individuals with BED also reported a decreased parasympathetic response, after a negative film mood induction (Svaldi et al., 2010) or a stress inducing procedure (Friederich et al., 2006), while other studies did not show differences (Hilbert et al., 2011). Future research should look at moderating, transdiagnostic factors, such as impulsivity, alexithymia, and emotion dysregulation that may explain mixed findings in the literature (Westwood et al., 2017; Hasking, 2019).

Average number of SCR and SCL levels increased for all participants, but there were no differences between groups or interaction effects. Skin conductance has generally been thought to be a measure of arousal that increases during positive and negative emotions (Alpers et al., 2011). Therefore, it would be expected—as found in this study—that increased emotional reactivity would lead to increased average number of SCR and average SCL levels. Similar to our findings, there have been studies with participants with EDs that failed to show differences in SCL levels compared to HCs after negative mood induction or exposure to stress (Tuschen-Caffier and Vogele, 1999; Svaldi et al., 2010; Hilbert et al., 2011).

### Strengths, Limitations, and Conclusions

High BMI and associated medical conditions may cause blunted cardiovascular responses (Masi et al., 2007; Carroll et al., 2012), which may have lowered RSA levels in the BED group. While our sample size was larger than most studies published to date using ED samples with similar measures, the overall sample size was relatively small. Some participants may have been familiar with the film stimuli, which may have allowed them to predict what would happen next and lead potentially to blunted psychophysiological responses. Memories of the context in which these movies were viewed in (e.g., to avoid the sound of family conflict) may have introduced noise in the collection of the psychophysiological data. Finally, since our study only included women, our results may not generalize to men.

Our study also possesses certain strengths. To our knowledge there is currently no other study that has used a mixed sample across EDs to record psychophysiological responses to a variety of standardized validated film clip types. Standardized validated film clips elicited expected affective responses and skin conductance responses in both ED and HC groups.

ED groups relative to individuals without EDs report more negative emotions generally, but were not more emotionally reactive to our film stimuli, which lacked ED-specific content. This has important treatment implications, as targeting non-ED specific cues may not be as effective at reducing ED pathology as reducing ED-specific cues. Throughout the protocol BED group had lower Respiratory Sinus Arrhythmia levels than the BN and HC groups. Respiratory Sinus Arrhythmia responses on this measure did not map onto the pattern of self-reported responses to the emotion-eliciting films presented, suggesting the importance of considering weight and cardiovascular problems in the use of this measure when assessing emotional response. The pattern of urges to binge for the different groups also did not match the pattern of self-reported emotional responses to the film clips. BN and BED groups experienced decreased urges to binge during all film clips compared to Baseline, suggesting that non-ED specific emotion-eliciting stimuli may at least temporarily decrease urges to binge, even while inducing negative affect. Future research is needed to understand the role of emotion-eliciting stimuli in reducing urges to binge in binge-eating groups.

## Data Availability Statement

The datasets presented in this article are not readily available because they contain protected health information. Requests to access the datasets should be directed to the corresponding author, EY Chen. The output of the results may be found in https://osf.io/8vxen/?view_only=73da0b3db29c47558f74133e8112f3a3.

## Ethics Statement

The studies involving human participants were reviewed and approved by University of Chicago Institutional Review Board and Temple University of The Commonwealth Institutional Review Board. The patients/participants provided their written informed consent to participate in this study.

## Author Contributions

MNF: writing—original draft and revising (lead), formal analysis (lead), and conceptualization (equal). EYC: study design, original research idea, and methodology (lead), conceptualization (equal), mentoring and supervision (lead), writing—review and editing (equal), and formal analysis (supporting). All authors contributed to the article and approved the submitted version.

## Conflict of Interest

The authors declare that the research was conducted in the absence of any commercial or financial relationships that could be construed as a potential conflict of interest.
