# Supplementary material for: Emotion and Psychophysiological Responses During Emotion–Eliciting Film Clips in an Eating Disorders Sample
Source: Front Psychol. 2021 Jul 8;12:630426. doi: 10.3389/fpsyg.2021.630426 (PMC8336872; doi:10.3389/fpsyg.2021.630426)
Supplement: Supplementary file 1 [file Table_1.DOCX]

Supplementary Material

***Supplementary Material 1***. Full Inclusion and Exclusion criteria for participation in study.

**Inclusions for eating disorder sample include**: being medically stable for outpatient assessment and treatment by an external medical exam; female; aged 18-60 years, meeting DSM-IV-TR (APA., 2000) diagnosis of Bulimia Nervosa or Binge Eating Disorder or International Classification of Diseases 10^th^ edition Typical or Atypical Anorexia Nervosa diagnoses (World Health Organization; WHO, 1992), with no current drug or alcohol dependence or symptoms of psychosis.

**Exclusions for the eating disorder sample**: past bariatric surgery or seeking bariatric surgery, in a weight loss intervention currently, or on medications primarily indicated for appetite or weight, e.g., Sibutramine. Other psychotropics, including antidepressants, were acceptable if the dose was stable for at least 3 months prior to screening. Healthy controls were eligible to participate if they did not meet diagnosis for current Axis I disorders (APA., 2000).

**Exclusions for both eating disorder and healthy control participants**: use of anti-seizure, beta-blockers, motion-sickness medications (e.g., Scopolamine) and asthma medication were excluded as these affect cardiac signals.

***Supplementary Table 2***. Correlation Coefficient Matrix for Demographic Variables

|  | BMI | Age | Poverty | Race |
| --- | --- | --- | --- | --- |
| BMI | **1.000** |  |  |  |
| Age | **0.31**** | **1.000** |  |  |
| Income-status †‡ | -0.35** | -0.343** | 1.000 |  |
| Race | 0.102 | -0.026 | 0.055 | 1.000 |

*Note.* There were 16 participants with Anorexia Nervosa, 57 with Binge Eating Disorder, 34 with Bulimia Nervosa, and 26 Healthy Controls.

**p < .05

The bolded values are Pearson's coefficients. All other correlations are Spearman's coefficients.

† Income status = low income status i.e., household income is < $25 000.

‡ *Note*. There were two participants for low income (1 BN, 1 HC)
